# The Broad Host Range and Genetic Diversity of Mammalian and Avian Astroviruses

**DOI:** 10.3390/v9050102

**Published:** 2017-05-10

**Authors:** Celeste Donato, Dhanasekaran Vijaykrishna

**Affiliations:** 1 Biomedicine Discovery Institute and Department of Microbiology, Monash University, Melbourne, Victoria 3800, Australia; celeste.donato@monash.edu; 2 Duke-NUS Medical School, Singapore 169857, Singapore

**Keywords:** astrovirus, classification, avian, mammal, genetic diversity, capsid, RNA-dependent-RNA-polymerase, ORF1b, ORF2

## Abstract

Astroviruses are a diverse family of viruses that infect a wide range of mammalian and avian hosts. Here we describe the phylogenetic diversity and current classification methodology of astroviruses based on the ORF1b and ORF2 genes, highlighting the propensity of astroviruses to undergo interspecies transmission and genetic recombination which greatly increase diversity and complicate attempts at a unified and comprehensive classification strategy.

## 1. Introduction

Astroviruses (AstVs) were first described in 1975 as small viruses that are 28–30 nm in diameter with icosahedral morphology. Astroviruses are named due to the distinct five-pointed or six-pointed star-like appearance of some virions when visualized under an electron microscope (EM); astrovirus is derived from the Greek word *astron* meaning star [1,2,3]. Astroviruses were first described from human infants with diarrhea and were subsequently identified in the young of numerous mammalian and avian species [4,5].

Human astroviurses (HAstVs) have been recognized as one of the major causes of acute gastroenteritis in children, associated with 2–8% of infections [6]. Transmission of HAstV occurs via the fecal-oral route, person-to-person contact, or contaminated food or water. Following an incubation period of 3–4 days, symptoms including diarrhea, vomiting, abdominal pain, and fever are often reported [7,8]. Whilst primarily associated with asymptomatic or diarrheal disease in humans, there are several reports of central nervous system (CNS) complications such as acute flaccid paralysis [9], meningitis, and encephalitis [10,11]. Animal astroviurses, have been isolated from numerous mammalian and avian species. In animals, astrovirus infection may be asymptomatic or associated with enteric disease and a range of other symptoms indicative of the involvement of other organ systems including hepatitis and nephritis in avian species [12,13], and neurological symptoms in cattle [14,15,16] and mink [17].

### 1.1. Genome

Astroviruses are classified within the unassigned *Astroviridae* family and are non-enveloped viruses characterized by a positive sense, single-stranded RNA (ssRNA) genome 6.4–7.9 kb long comprised of a 5′-untranslated region (UTR), three open reading frames (ORFs)—ORF1a, ORF1b, and ORF2, a 3′-UTR, and a poly A tail [18]. The ORF1a region encodes a non-structural polyprotein (serine protease), ORF1b encodes a polyprotein including the RNA-dependent RNA polymerase (RdRp), and ORF2 encodes the viral capsid protein [18]. A further ORF, termed ORFX, has been observed in classic HAstVs and some mammalian astroviurses, overlapping the 5′ end of ORF2 which may be translated through a leaking scanning mechanism [19]. Astroviruses exhibit several distinctive features in addition to a distinctive morphology. The viruses lack a RNA-helicase domain encoded within the genome and utilize a ribosomal frameshifting mechanism to translate the RdRp, which distinguishes the *Astroviridae* family from other non-enveloped ssRNA virus families such as *Picornaviridae* and *Caliciviridae* [20,21]. The greatest diversity in the genome is within the ORF2 region, which is characterized by a highly-conserved N-terminal domain (amino acids (aa) 1–424), a hypervariable domain (aa 425–688) which is believed to form the capsid spike and contribute to receptor binding, and a highly acidic C-terminal domain [22,23].

### 1.2. Classification

The wide host range of astroviruses and the high degree of genetic diversity present within the *Astroviridae* family have complicated attempts at a unified classification method. Astroviruses are classified within the unassigned *Astroviridae* family, which was initially comprised of a single genus (*Astrovirus*) based on virion morphology [18]. The classification of the *Astrovirus* genus within the family *Astroviridae* was recognized by the International Committee for Taxonomy of Viruses (ICTV) in 1995 and the classification scheme has been modified numerous times over the intervening years [24]. In 2004, two genera were recognized; *Mamastrovirus* (MAstV) and *Avastrovirus* (AAstV), that were known to infect mammalian and avian species, respectively, and viruses were classified solely on the species of origin [24]. However, the advent of sequence based characterization rendered this approach inadequate, revealing that viruses isolated from different species can be genetically similar (reflecting prevalent interspecies transmission of viruses) and also revealing a large range of diversity of viruses within a single host species. With this in mind, the classification system proposed by the ICTV *Astroviridae* Study Group in 2010 recommended classification based on the amino acid sequence of the ORF2 genome region, recommending that different strains of the same astrovirus species should share >75% identity in the capsid proteins [25]. Additionally, there are proposals to define distinct variants within a recognized astrovirus species, with a variant defined as sharing <93–95% nucleotide similarity to the reference or prototype strain of each species or if phylogenetic analysis is used, a distance of >0.05 based on analysis of the capsid protein [12,18].

**Figure 1:**
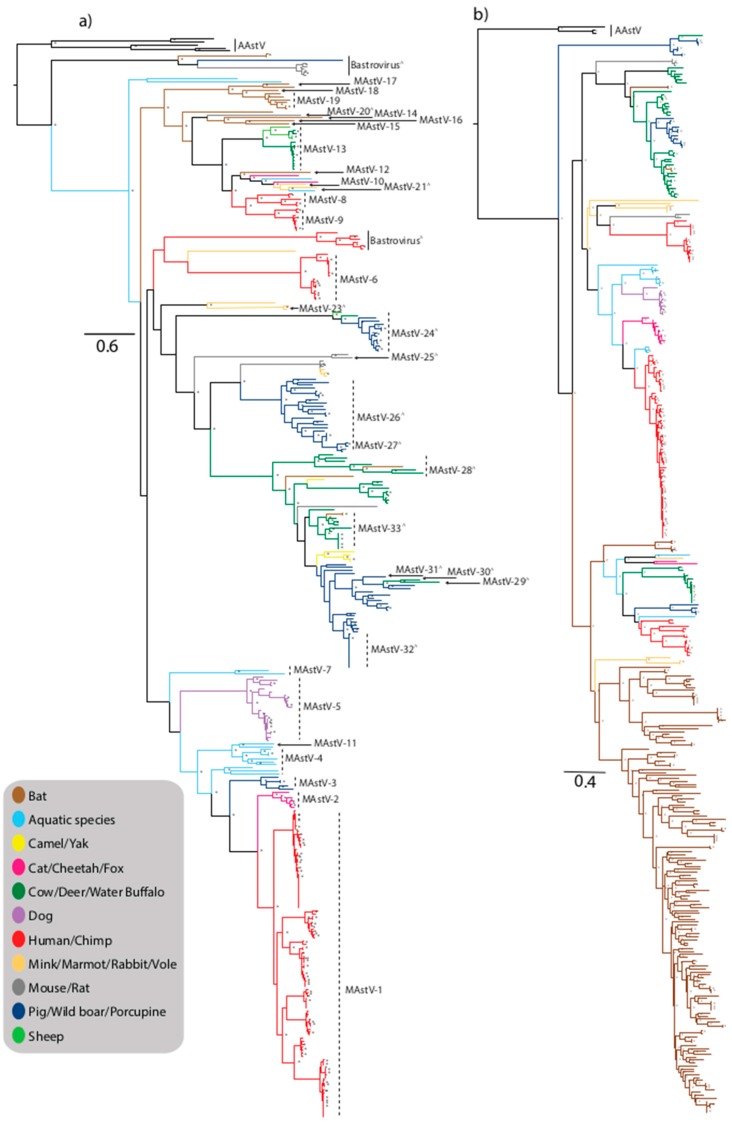
Maximum-likelihood phylogenetic tree of *Mamastrovirus* (MAstV) capsid (**a**) and RNA-dependent RNA polymerase (RdRp) (**b**) genes. Trees were generated from nucleotide sequences using the maximum-likelihood method with the general time reversible nucleotide substitution model with gamma distribution (GTRG+G) and 1000 bootstrap replicates and nodes with bootstrap support values ≥70 are shown by an asterisk. Proposed species yet to be recognized are designated with a ^ symbol. Strains are colored by host and trees with full taxa names are provided as Supplementary Materials (Figures S1 and S2).

Astroviruses within the *Mamastrovirus* genus are derived from numerous mammalian species in addition to humans (HAstV), including farmed species such as pigs (PAstV), sheep (OAstV), cattle (BoAstV), domesticated animals including cats (FAstV), and dogs (CaAstV), rodents and small mammals including mink (MiAstV), bats (BAstV), rats (RAstV), mice, rabbit (RabAstV), fox, marmot (HHMastV), porcupine, shrew, vole, and larger species including deer (CcAstV), monkeys, water buffalo (BufAstV), yak, camel (DcAstV), and cheetah (ChAstV) (Figure 1a,b). Viruses from the *Mamastrovirus* genus have also been characterized from marine mammals including stellar sea lion (SslAstV) and California sea lions (CslAstV), minke whale, orca whale, and bottlenose dolphins (BdAstV) (Figure 1a,b) [26]. The current ICTV classification reveals 19 recognized species of *Mamastrovirus* (MAstV-1–19) within two genogroups GI and GII; *Mamastrovirus 1* (GI.A-human); *Mamastrovirus 2* (GI.B-feline); *Mamastrovirus 3* (GI.C-porcine); *Mamastrovirus 4* (GI.D-California sea lion); *Mamastrovirus 5* (GI.E-canine); *Mamastrovirus 6* (GI.F-human); *Mamastrovirus 7* (GI.G-bottlenose dolphin); *Mamastrovirus 8* (GII.A-human); *Mamastrovirus 9* (GII.B-human); *Mamastrovirus 10* (GII.C-mink); *Mamastrovirus 11* (GII.D-California sea lion), *Mamastrovirus 12* (GII.E-bat); *Mamastrovirus 13* (GII.F-ovine); and *Mamastroviruses 14–19* (GII.G to GII.L-bat species), and numerous other strains awaiting classification, some of which are considered as tentative new species (designated by a ^∧^ symbol in the phylogenetic trees) (Figure 1a) [27,28].

**Figure 2:**
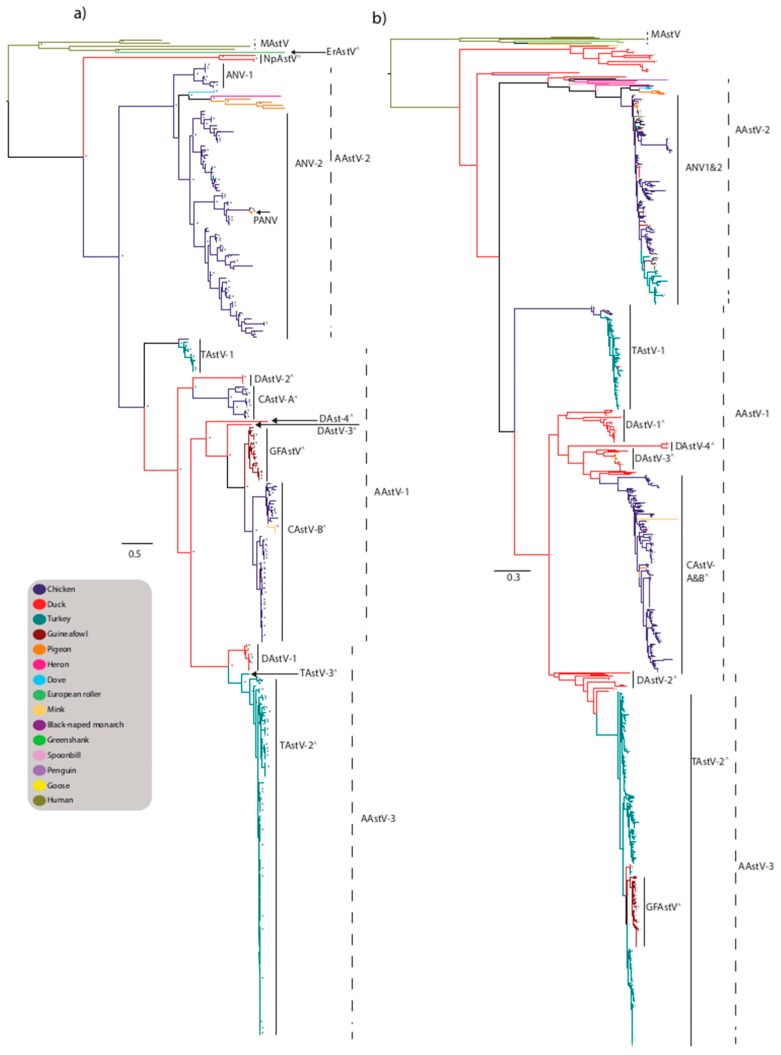
Maximum-likelihood phylogenetic tree of *Avastrovirus* (AAstV) (**a**) capsid and (**b**) RdRp genes. Trees were generated from nucleotide sequences using the maximum-likelihood method with the GRT+G nucleotide substitution model and 1000 bootstrap replicates and nodes with bootstrap support values ≥70 are shown by an asterisk. Proposed species yet to be recognized are designated with a ^ symbol. Strains are colored by host and trees with full taxa names are provided as Supplementary Materials (Figures S3 and S4).

Viruses from the *Avastrovirus* genus have been characterized from numerous farmed avian species including turkeys (TAstV), ducks (DAstV), chicken (CAstV), guineafowl (GFAstV), pigeon (PiAstV), goose, as well as wild aquatic and terrestrial birds including heron, doves, penguins, and many other species (Figure 2a). The three species originally recognized within the genus were *Avastrovirus GI.A* comprised of turkey astrovirus 1 (TAstV-1), *Avastrovirus GI.B* comprised of avian nephritis virus 1 (ANV-1), avian nephritis virus 2 (ANV-2), and *Avastrovirus GII.A* comprised of turkey astrovirus 2 (TAstV-2) and duck astrovirus DAstV/C-NGB [25]. *Avastrovirus GI.A*, *Avastrovirus GI.B*, and *Avastrovirus GII.A* were renamed *Avastrovirus 1* (AAstV-1), *Avastrovirus 2* (AAstV-2), and *Avastrovirus 3* (AAstV-3), respectively [25].

Currently, classification into species is based on the phylogenetic analysis of the amino acid sequence of the full length ORF2 region of the genome that encodes the capsid. However, the limited number of capsid sequences available compared to RdRp sequences makes consistent classification difficult, especially with some novel viruses incompletely sequenced. There are numerous unclassified astroviruses, particularly isolated from aquatic and terrestrial wild birds which, according to the ICTV, are “related viruses which may be members of the *Avastrovirus* genus but have not been approved as species” [18].

## 2. Human Astroviruses

Astrovirus infection in humans has been primarily associated with diarrhea and vomiting, accounting for up to 10% of sporadic gastroenteritis cases in some regions [29]. CNS complications associated with astrovirus infection have been reported in recent years, including acute flaccid paralysis, with some fatalities reported in children with underlying immune disorders [30,31].

Historically human astroviruses (HAstV) were classified into five serotypes in 1984 [32]. Subsequent molecular characterization based on viral reactivity to polyclonal antibodies and nucleotide sequence analysis led to the recognition of eight serotypes (HAstV-1–8), now termed “classic” HAstV [18,33,34]. The relatively recent advent of next generation sequencing (NGS) and metagenomic analysis has led to the identification of numerous novel strains considered “non-classic” HAstV [18]. Currently, HAstVs are classified within the species MAstV-1 (HAstV-1–8), MAstV-6 (MLB1–3), MAstV-8 (VA2/HMO-A, VA4, VA5, BF34), and MAstV-9 (VA1/HMO-C, VA3/HMO-B) [18] (Figure 1a).

### 2.1. MAstV-1

The MAstV-1 species is comprised of HAstV-1–8, and surveillance has revealed that HAstV-1 is the most commonly detected type in children, followed by HAstV-2–5, whereas HAstV-6–8 have been rarely detected [35]. HAstV-4 and HAstV-8 have been associated with infection of older children and longer duration of diarrhea (>7 days) [36,37]. A HAstV-4 strain was also isolated from an infant with fatal meningoencephalitis [30]. Based upon the phylogenetic analysis of the ORF2 region, different lineages within each HAstV type have been proposed; HAstV-1 (HAstV-1a–d) and HAstV-2 (HAstV-2a–d) have been divided into four lineages, whereas HAstV-3 (HAstV-3a–b) and HAstV-4 (HAstV-4a–c) have been classified into two and three lineages, respectively [38].

### 2.2. MAstV-6

The first “non-classic” HAstV strain characterized was MLB1, the virus was detected in a stool sample from a 3 year old Australian child with acute diarrhea in 1999; the child had previously received a liver transplant [39]. The majority of MLB1 strains characterized to date have been detected in India, Kenya, and Japan with limited detected in the USA, China, Bhutan, Egypt, Brazil, and Italy and prevalence has been reported in the range of 0.2% to 9% [40]. However, a seroepidemiologic study in the USA revealed that primary exposure to MLB1 occurs in childhood and that seropositivity reached 100% by adulthood suggesting the widespread circulation of the virus in the human population [41]. MLB2 viruses were first identified in Vellore, India [42] with the majority of strains subsequently identified in Japan, The Gambia, and Switzerland with limited detection in Turkey, USA, Kenya, China, and Thailand and prevalence reported in the range of 0.3% to 1.5% [40]. MLB2 has been associated with meningitis and other CNS complications and has been detected in immunocompromised children [10]. MLB3 viruses were first detected in India in 2004 [43], with subsequent detection in Kenya and The Gambia and the prevalence in stools ranges from 0.6% to 3.1% [40,44].

### 2.3. MAstV-8

There is a dual naming system for some HAstV species due to the simultaneous characterization of these viruses by different researchers; these viruses are termed VA/HMO named for VA—Virginia and HMO—Human-Mink-Ovine-like viruses, due to their genetic relatedness to previously characterized mink and sheep viruses [9]. In 2009, VA2/HMO-A strains were detected in children with non-polio acute flaccid paralysis in Nigeria, Pakistan [9], and India [42]. The prevalence of VA2/HMO-A viruses in stools ranges from 0.3% to 2.3%, with strains also detected in Egypt, Japan, USA, Kenya, and China [40]. VA4 has only been detected in Nepal [43] and the BF34 strain has only detected in Burkina Faso [45].

### 2.4. MAstV-9

The VA3/HMO-B viruses were first identified in Vellore, India [42] with sporadic detection in Nigeria, Pakistan, and Nepal with prevalence ranging from 0.1% to 2.3% [40]. VA1/HMO-C viruses were first detected in 2009 during an outbreak of diarrhea in children in Virginia, USA [46]. VA1/HMO-C viruses have been detected and associated with encephalitis in immunocompromized children and adults [11,40,47] and acute respiratory disease [48]. The prevalence of VA1/HMO-C viruses in stools ranges from 0.2% to 1.6% in diarrheic and non-diarrheic subjects worldwide, with limited detection in Nepal, Japan, Tanzania, The Gambia, France, and the U.K. [9,44,47]. Seroprevalence of VA1/HMO-C has been reported at 65% in adults [49].

### 2.5. Bastrovirus

The divergent human astrovirus-like virus tentatively named Bastrovirus was isolated from patients in The Netherlands. The capsid region is homologous to the capsid of HAstV whilst the RdRp region is more closely related to members of the *Hepeviridae* family. This virus remains to be classified by the ICVT, however the capsid regions clusters closest to the MLB1-3 viruses suggesting an evolutionary relationship to these divergent human astroviruses [50] (Figure 1a). Revealing the geographic and host diversity of this virus, divergent Bastrovirus strains have also been isolated from bat, pig, and rat species in Vietnam, forming a distinct cluster of strains that also await classification (Figure 1a).

## 3. Non-Human MAstV

*Mamastroviruses* are capable of infecting a wide range of mammalian species including companion animals (cats and dogs), intensively farmed species (pigs and cattle), as well as terrestrial and aquatic wild mammalian species. Unexpectedly there is no clear clustering of viruses separated by hosts of terrestrial or aquatic origins (Figure 1a,b). In addition to the classified MAstV species, there are numerous divergent viruses isolated from diverse species which likely represent new species awaiting formal classification (Figure 1a) [51].

Not surprisingly, domesticated animals such as cats and dogs harbor astrovirus strains more closely related to HAstV than the viruses harboured by many other animal species. Feline astrovirus was first identified in 1981, and feline strains form a small discrete cluster defined as the species MAstV-2, closely related to the human strains comprising the species MAstV-1 [52] (Figure 1a). Based on evolutionary analysis, an interspecies transmission pathway has been hypothesised whereby porcine strains may have been transmitted to cats and subsequently to humans, possibly involving other intermediary species suggesting sustained interspecies transmission events [53]. Characterization of an outbreak of diarrhea in a group of captive cheetahs in a breeding facility identified an astrovirus strain most closely related to feline strains (Figure 1a), however it is not clear if this is a recent transmission from domesticated cats or if the virus is circulating independently in cheetahs [54]. Despite dogs also being a companion animal, canine astrovirus, first described in the 1980s, are not as closely related to human strains as feline strains appear to be, based on phylogenetic analysis (Figure 1a,b). Canine strains form a small, discrete cluster comprising species MAstV-5, within a lineage comprised of dolphin and sea lion clades representing species MAstV-4 and -11 [55,56], and feline, porcine, and human strains are more distantly related within the same lineage (Figure 1a).

A large diverse lineage, largely comprised of unclassified viruses or tentatively classified viruses awaiting approval encompasses divergent marmot and rabbit strains (MAstV-23), small, discrete clusters of porcine/ovine/bovine strains (MAstV-24), divergent rat strains (MAstV-25), and unclassified mouse and mink strains forming a small discrete cluster (Figure 1a). Numerous porcine strains also cluster within this lineage; PAstV comprise *Mamastrovirus 3* and were first detected by EM in pigs in the U.K. and the USA [57], and are now recognized to have worldwide distribution [12]. There is a high prevalence of astrovirus detection in pigs, up to 80% in some studies, suggesting that pigs may be persistently infected with various strains of PAstV [58]. There are five recognized lineages (PAstV1–5) reflecting the diversity of strains, suggesting swine are highly permissive to astrovirus infection often without symptoms of disease (Figure 1a,b). This diversity likely reflects the varying origins of these viruses, in particular highlighting frequent interspecies transmission and recombination events [58,59,60]. A study in the USA revealed the high level of co-infections in pigs (13.9%), revealing the frequent opportunity for recombination, especially between viruses of different lineages [61]. The close clustering between some porcine and humans strains, in particular MAstV-3 and MAstV-1 viruses, reflects the close contact between these species with the close sharing of environments and co-housing documented in some countries facilitating frequent interspecies transmission [62]. Wild boars showing no symptoms of enteric disease, housed in a captive breeding park with no contact with domesticated pigs, were found to harbor astrovirus strains with a high degree of genetic similarity to porcine strains comprising MAstV-26 and suggests that the virus may be derived from commonly circulating porcine strains [63] (Figure 1a,b). Porcupine strains isolated in China also cluster with unclassified PAstV-2 strains suggesting further interspecies transmission of these viruses (Figure 1a) [64].

Unexpectedly, limited astrovirus strains have been isolated from sheep. Ovine astrovirus was first identified by EM in 1977 [4], and OAstV-1 clusters with bovine strains comprising MAstV-13 and a second strain from a healthy sheep characterized in 2009, OAstV-2, clusters with porcine and bovine strains comprising MAstV-24 [65] (Figure 1a). Similarly, astroviruses are not associated with a significant burden of diarrheal disease in bovine species. The first bovine astrovirus was detected in England in 1978 [66] and bovine astrovirus strains have been detection in association with neurological disease, including encephalitis (BoAstV-CH13/NeuroS1) [14,15,16,67,68,69] and diarrheal disease in calves in South Korea [70] and cattle and buffalo calves in China [71]. Two serotypes were previously recognized, BoAstV-1 and BoAstV-2 [72], however based on phylogenetic analysis there are multiple lineages of BoAstV strains circulating in farmed bovine populations, and the close clustering of bovine, porcine, and ovine strains in multiple lineages reflects the common interspecies transmission events that occur between farmed animals (Figure 1a,b). Phylogenetic analysis also reveals that bovine-like astrovirus strains have been isolated from numerous wild species including water buffalo, yak, and European roe deer (CcAstV-1 and CcAstV-2) suffering from gastroenteritis [73]. Unclassified astroviruses from dromedary camels (DcAstV) [27] also cluster in a lineage comprised of porcine and bovine strains, further suggesting multiple interspecies transmission events (Figure 1a).

Substantial astrovirus strain diversity has been observed in small mammals, primarily rodents and bats, forming both species-specific clusters reflecting endemic transmission and co-clustering of strains with other host species reflecting widespread interspecies transmission (Figure 1a,b). Novel murine astrovirus strains have been isolated from laboratory mice in the USA and Japan [74,75]. Divergent viruses have also been detected, such as those detected in rats (MAstV-25), highlighting the need for more detailed detection and characterization of these viruses to better understand the role these animals play in astrovirus transmission between varied species. One of the mammalian species with the highest burden of symptomatic astrovirus infection is mink; infection is associated with pre-weaning diarrhea syndrome and the neurological condition “shaking mink syndrome” [17,76]. Mink viruses cluster within multiple lineages suggesting that the species is permissive to infection with multiple, diverse lineages of viruses (Figure 1a). Bat astroviruses were first detected in 2008 in Hong Kong [77] and subsequently detected in bats in China [78,79], North America [80], Germany [81], Hungary [82], and the Czech Republic [83] from numerous bat species, with detection rates ranging from 36% to 100% in *Miniopterus magnater* bats and from 50% to 70% of *Miniopterus pusillus* bats sampled in Hong Kong [77]. A diverse population of viruses appears to be highly prevalent in bats without causing disease [12] (Figure 1 and Figure 2). The majority of bat astroviruses are divergent from other characterized mammalian astroviruses, and display a high degree of genetic diversity forming numerous recognized and proposed species (Figure 1a). Some bat sequences clustered with strains from other species including fox, cattle, and mice, suggesting that bats are highly permissive to infection with diverse astrovirus strains from multiple hosts and play a key role in astrovirus diversity and interspecies transmission (Figure 1a,b).

Astroviruses have been detected in aquatic mammalian species including Californian sea lions, Steller sea lion, bottlenose dolphin, killer whale, and minke whale [26]. The strains from these aquatic species do not cluster together, instead forming multiple discrete clusters, suggesting several transmission events from terrestrial mammals to aquatic mammals (Figure 1a). Minke whale and BdAstV-3 strains are divergent to characterized astrovirus strains, with BdAstV-1 strains comprising MAstV-7 and a diverse group of sea lion viruses comprising the MAstV-4 cluster with porcine and canine strains (Figure 1a).

There are numerous divergent strains that are yet to be classified that may reflect interspecies transmission events. A single divergent feline strain along with a fox strain cluster within a diverse lineage comprised of human HMO strains (MAstV-8 and -9), sea lion (MAstV-11), unclassified sea lion, mink (MAstV-21), and bat (MAstV-12) strains. A Himalayan marmot strain clusters with MAstV-6 strains (Figure 1a,b). There appears to be a large diversity of MAstV strains not captured by the currently available sequences, highlighting the need for more detailed sampling and characterization of animal strains.

## 4. Avastrovirus

The isolation of astroviruses from avian species predates their isolation in humans, with disease in ducklings described in 1965, however the virus was not formally recognized as an astrovirus until 1984 [5,84]. Avian astroviruses have been documented to cause infection in poultry leading to economic losses in farms and affecting food production worldwide [85]. Avian astroviruses have been associated with a spectrum of disease ranging from subclinical infection in seemingly healthy adult birds to heavy flock losses. Pleomorphic symptoms include enteritis in turkeys, chickens, and guineafowl, mild growth depression and nephritis in chickens, and hepatitis in ducklings [86]. Astrovirus infection has been implicated in pre-hatching mortality in ducklings and goslings [87].

In addition to AAstV-1 comprised of TAstV-1, AAstV-2 comprised of ANV1 and ANV2, and AAstV-3 comprised of TAstV-2 and duck astrovirus 1 (DAstV-1), there are numerous yet-to-be classified viruses, including Turkey astrovirus 3 (TAstV-3), chicken astrovirus A (CAstV-A), chicken astrovirus B (CAstV-B) [88,89], GFAstV, and multiple viruses isolated from ducks including duck hepatitis virus 3 (DHV-3/DAstV-2) [88], DHV-3/DAstV-2-like viruses, DAstV-CPH (DAstV-3) [90], DAstV-4, DAstV-YP/DAstV-4-like, and diverse viruses isolated from wild birds [86].

### 4.1. TAstV

Avian astrovirus have been detected in outbreaks of enteric disease in turkey poults, and avian astrovirus were first reported as an agent of gastroenteritis and mortality in young turkeys in 1980, associated with a condition known as poult enteritis mortality syndrome (PEMS) [91,92]. Subsequently there have been sporadic reports of astrovirus outbreaks in turkeys, associated with enteritis and growth depression [93]. Based on serological and genetic analysis, two types of TAstV have been recognized (TAstV-1 and TAstV-2). TAstV-1 comprises the *Avastrovirus 1* species and was first described in the U.K. [91]. TAstV-1 has limited detection in other avian species with sporadic detection in chicken and ducks and forms a discrete cluster in both the capsid and RdRp phylogenetic analysis (Figure 2a,b). TAstV-2 was identified in 1996 [94], and is likely to be classified within the species *Avastrovirus 3* and is primarily associated with PEMS [95]. TAstV-2 is the predominant TAstV lineage, with a wider global circulation and greater genetic diversity compared to TAstV-1, reflected by the co-circulation of multiple sub-lineages (Figure 2a,b). Based on phylogenetic analysis of the RdRp gene there appears to be limited interspecies transmission of TAstV-2-like viruses detected in chicken and duck (Figure 2b). Astroviruses infecting guineafowl are closely related to TAstV-2 strains based on analysis of the RdRp region [96], however the capsid region is distinct to TAstV-2 strains, forming a discrete cluster in a lineage of unassigned viruses suggesting they are closely related to CAstV-B strains (Figure 2a). These GFAstVs were possibly derived from recombination and interspecies events followed by sustained transmission in the guineafowl population. This highlights the limitation of classifying viruses based on a single region of the genome. 

### 4.2. CAstV

Chicken astrovirus has been associated with runting-stunting syndrome (RSS) in chickens characterized by poor weight gain, lower feed conversion, and mortality resulting in economic losses [97], and “white chicks” disease associated with increased mortality of embryos and chicks, weakness, and white plumage [98]. Currently two serotypes of CAstV have been described [97], and both serotypes form discrete clusters in the phylogenetic analysis of the capsid region within a lineage of unclassified viruses (likely to be classified within AAstV-1). The CAstV-A viruses form a smaller, discrete lineage, clustering closest to DAstV-2 strains (Figure 2a). CAstV-B strains form a large lineage clustering closest to GFAstV strains. Based on phylogenetic analysis of the small region of the RdRp gene commonly sequenced, it does not allow CAstV-A and -B strains to form discrete clusters as seen in the capsid analysis, possibly reflecting multiple recombination events in CAstV strains (Figure 2b). CAstV strains have limited detection in other avian species with sporadic detection in pigeon and duck (Figure 2a,b).

### 4.3. ANV

Avian nephritis virus was identified in association with intestinal nephritis in chickens and growth retardation. The first serotype of ANV (ANV-1) was isolated from a healthy broiler chick in 1976 [99] and was initially regarded as a picornavirus, subsequently reclassified within the *Astroviridae* family in 2000 [100]. Based on capsid phylogenetic analysis, ANV-1 strains cluster within the AAstV-2 lineage forming a small, discrete, relatively conserved cluster (Figure 2a). The second serotype (ANV-2) was later described from chicks with stunted growth [101]. Based on capsid phylogenetic analysis, ANV-2 strains cluster within the AAstV-2 lineage, forming a larger, more diverse sub-lineage compared to ANV-1. ANV-1 and ANV-2 viruses have also been sporadically detected in ducks and turkeys (Figure 2a). A third serotype (ANV-3) detected in chickens and turkeys with RSS and locomotion impairment has been proposed based on sequencing of the ORF1a region, however complete genome sequences are unavailable for adequate comparisons to the recognized serotypes [102]. The virus designated pigeon ANV (P-ANV) was detected during an outbreak of gastrointestinal illness in young pigeons in China [103]. P-ANV represents an interspecies transmission event from chickens to pigeon; based on phylogenetic analysis, P-ANV strains share a high degree of genetic similarity to ANV-2 strains and cluster closely with strains also circulating in China suggesting a localized transmission event, and these viruses should be considered as ANV-2 viruses and do not require a distinct designation (Figure 2a) [104]. Based on phylogenetic analysis of the small region of the RdRp gene commonly sequenced, it does not allow ANV 1 and 2 strains to form discrete clusters as characterized in the capsid analysis (Figure 2b). Whilst this may reflect common recombination events between ANV strains, the analysis of a small region does not adequately allow for distinct clustering. 

### 4.4. DAstV

Astrovirus infection in ducks has been associated with a highly contagious and fatal hepatitis, historically known as duck hepatitis virus type 2 (DHV-2), which was described in the U.K. and subsequently serotype DHV-3 was isolated in the USA [5,84,105,106]. The 9th ICTV report classified DHV-2 and DHV-3 as DAstV-1 and DAstV-2, respectively [25]. DAstV-1 strains form a small, discrete cluster within the AAstV-3 lineage based on phylogenetic analysis of the capsid region, clustering closest to TAstV-2 strains (Figure 2a). DAstV-2 viruses, currently unclassified, form a discrete, highly conserved cluster closest to CAstV-A strains (Figure 2a). Based on phylogenetic analysis of the RdRp region, DAstV-1 is closely related to other unclassified duck strains whilst DAstV-2 strains are more closely related to TAstV-2 strains (Figure 2b). Further unclassified serotypes have been described; DAstV-3-CPH, DAstV/C-NGB, and DAstV-4 [107]. Duck astrovirus DAstV-3/CPH has been suggested to transmit horizontally and vertically [108] and DAstV-3/CPH viruses have also been detected in goslings [107]. Phylogenetic analysis of the RdRp region highlights the diversity of AAstV strains circulating in duck species (Figure 2b). Strains endemic to ducks have limited detection in other avian species including geese (Figure 2a,b).

### 4.5. Novel Astroviruses Detected in Wild Birds

Astroviruses have been detected in numerous wild aquatic species including teals, pintails, and shovelers (belonging to the order Anseriformes), sanderlings (order Charadriiformes), and herons and spoonbills (order Pelecaniformes) [86,109]. Fewer land dwelling wild birds have been found to harbor avian astroviruses including doves and pigeons (order Colombiformes), European roller (order Coraciiformes), and black-naped monarch (order Passeriformes) [51,104,110,111]. These viruses are highly divergent and largely unclassified, suggesting these viruses are endemic to the wild bird population (Figure 2a,b). The migratory behavior of some of these species provides ideal conditions for virus dissemination and diversification as during migration there is a high degree of co-mingling and increased density of birds of different species that may originate from varied geographic regions [112].

## 5. Interspecies Transmission

Phylogenies indicate that the genetic diversity of mamastroviruses has been shaped by extensive interspecies transmission events that have occurred in the past between wild and domestic species and humans. However, the inference of astrovirus interspecies transmission events is hampered due to (a) sequencing of only a small portion of the genome; (b) inadequate sampling; or (c) that the event occurred early during the divergence of astrovirus lineages. A few exceptions where host-jumps are apparent have been in livestock where a greater level of sampling has occurred. This includes hosts with a greater level of interaction (e.g., MAstV-13 in ovine and bovine) or host genetic similarly (e.g., MAstV-26 in wild boars and domestic pigs). With the exception of HAstV-4 strains associated with fatal meningoencephalitis, it is interesting to note that viruses from multiple species, all recognized to cause neurological symptoms, are closely related including human VA1/HMO-C viruses and mink and bovine viruses also associated with neurological symptoms, suggesting that these related viruses may have a distinct phenotype compared to other MAstV strains (Figure 1a,b). Astrovirus strains identified from fecal samples of multiple non-human primate species from wild, captive, and peri-urban environments in Bangladesh and Cambodia reveal multiple interspecies transmission events, with viruses closely related to the VA/HMO lineage of human viruses, and non-human mammalian and avian astroviruses (Figure 1a,b) [28].

Similarly, there appears to be evidence for a high degree of cross species transmission of avian astroviruses between farmed poultry species as described in the above sections. There also appears to be transmission between avian and mammalian species. The highly divergent strains isolated from European roller (Er/SZAL6/HUN/2011 and Er/BMTK529/HUN/2011) exhibited low identity to avian and mammalian astroviruses, cluster within the MAstV lineage, and were likely derived from multiple recombination and interspecies transmission events [51]. The carnivorous diet of this avian species may have facilitated the interspecies transmission event between a rodent or small mammal species. This is the only report of a *Mamastrovirus* strain in an avian species; in contrast, there have been more reports of *Avastrovirus* strains detected in mammalian species which may reflect greater sampling density of the mammalian population. A highly divergent group of mink strains detected in China represent a novel clade of astroviruses that were distantly related to previously described mink astrovirus and were closely related to chicken and turkey astroviruses (Figure 2a,b) [113]. These viruses are recombinant strains with the capsid region clustering with CAstV-B strains and the RdRp region clustering with human MAstV strains and CAstV strains (Figure 2a,b). There have been two *Avastrovirus* strains detected in humans; a strain clustering close to ANV-1 strains detected in turkey and chicken was isolated from a child in The Gambia, and a strain clustering with ANV-2 strains detected in chicken was isolated from a child in Kenya [44]. Serological studies have been used to screen human sera from poultry workers for antibodies to TAstV-2, with up to 26% of participants positive with the highest detection in abattoir workers and turkey growers [114], suggesting avian strains may be readily transmitted to humans under prolonged close contact. 

The important role that ecotones play in astrovirus cross-species transmission has been proposed [112]. Ecotones are ecological transition areas such as small and medium sized farms which rear multiple species. The co-rearing of poultry such as domestic ducks, chickens, turkey, and guineafowl can facilitate transmission between these species but also transmission to wild birds [112]. Farms and abattoirs have also been recognized as environments facilitating transmission between livestock species and to farm and abattoir workers [114]. Many other species have contact with livestock in a farming environment; in addition to wild species, companion animals such as cats and dogs and other peri-domestic animals have contact with livestock and their biological waste providing substantial opportunities for cross-species transmission. Astroviruses also persist in bodies of water making the aquatic environment ideal for the transmission of viruses infecting avian species, aquatic mammalian species, and possible transmission between terrestrial and aquatic species. Untreated or inadequately treated sewage and waste water from domestic and farmed areas can reach fresh and marine bodies of water transmitting human and animal viruses. Astroviruses have been detected in the environment and the durability of the virus in this environment may greatly contribute to cross-species transmission within and between terrestrial and aquatic species, generating significant diversity [115]. 

## 6. Recombination

In addition to interspecies transmission which generates significant diversity in astrovirus species, both intra-species and inter-species recombination can rapidly generate novel, divergent viruses. Full- and partial-genome sequence analysis has identified multiple strains that have undergone recombination events, which are predominately located within the ORF1b/ORF2 junction region of the genome, which is a region with an RNA secondary structure predicted to contain a stable hairpin structure [21,34,116,117,118,119]. A virus with a recombination event within the ORF1a region has been identified [119]. Some recombinants appear to be highly stable and show widespread detection [21], whilst others are detected sporadically as single strains. Numerous human recombinant strains have been reported between HAstV strains, including strains with HAstV-1 (ORF1b) and HAstV-4 (ORF2) parental viruses, HAstV-1 (ORF1b) and HAstV-3 (ORF2) parental viruses, HAstV-3 (ORF1b) and HAstV-2 (ORF2), and VA2 (ORF1b) and MLB1 (ORF2) parental viruses [116,119,120,121]. 

Divergent species often represent recombination events between strains of different species. In 2010, the study conducted by Rivera et al. suggested the possibility of a recombination event between human and California sea lion astrovirus strains [26]. A recombinant strain derived from porcine astrovirus and human HAstV-3 strains was reported from piglets and children from various regions of Colombia [62]. ANV strains that appear to be derived from recombination events between ANV-1 (ORF1b) and ANV-2 (ORF2) have been described in the USA [85].

## 7. Future Considerations for Classification

The increased sampling density of numerous host species combined with the more prevalent use of NGS technologies and viral metagenomic studies will increase the detection of novel strains, further driving the need for a unified, complex, and encompassing classification system. The previously common practice of sequencing a relatively conserved RdRp amplicon of 300–400 bp renders many sequences available in GenBank of little use in detailed phylogenetic analysis, as does the frequent missing metadata regarding species of isolation, country, and date of collection. Sequencing small regions is not adequate to determine if a virus strain is a novel, divergent strain or a recombinant virus. Recommendations should be made to encourage full genome sequencing where possible and the deposition of associated host and demographic information. Although analysis of amino acid sequences of the capsid region is required for classification, it leads to confusion regarding appropriate phylogenetic analysis, with highly inconsistent publication of nucleotide and amino acid trees further complicating attempts to clarify diversity and classification. The topologies of amino acid trees differ to those of nucleotide trees, particularly for the analysis of MAstV, and whilst amino acid trees are required for classification, nucleotide trees may be more appropriate for describing within species diversity (Figures S1 and S2). Incorporating a standardized nomenclature to aid in classification has proven invaluable in the classification of numerous viruses, including rotavirus and influenza [122]. Adopting a nomenclature that records the appropriate metadata associated with sample collection including host, location, date of collection, and determined species and serotype as proposed by Martella and colleagues would vastly improve the usability of strains for more complex analyses [38].

## Supplementary Materials

The following are available online at www.mdpi.com/1999-4915/9/5/102/s1, Figure S1: Maximum-likelihood phylogenetic tree of MAstV (**a**) capsid nucleotide and (**b**) capsid amino acid sequences, Figure S2: Maximum-likelihood phylogenetic tree of MAstV RdRp, Figure S3: Maximum-likelihood phylogenetic tree of AAstV (**a**) capsid nucleotide and (**b**) capsid amino acid sequences, Figure S4: Maximum-likelihood phylogenetic tree of AAstV RdRp.
