# Supplementary material for: The Broad Host Range and Genetic Diversity of Mammalian and Avian Astroviruses
Source: Viruses. 2017 May 10;9(5):102. doi: 10.3390/v9050102 (PMC5454415; doi:10.3390/v9050102)
Supplement: Supplementary file 1 [file viruses-09-00102-s001.zip › viruses-190761_final_supplementary/FigureS2.pdf]

S2

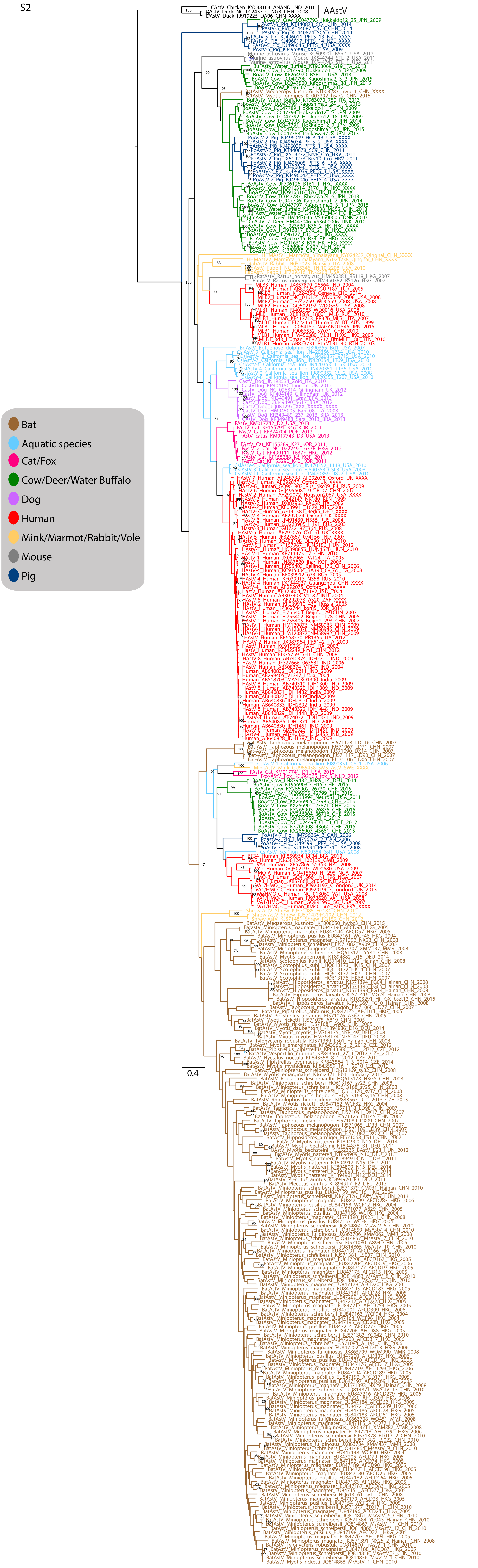

S2: Maximum-likelihood phylogenetic tree of MAstV RdRp
Trees were generated using the maximum-likelihood method with 1,000 bootstrap replicates and nodes with bootstrap support values ≥70 are shown. Proposed species yet to be recognized are designated with a ^ symbol. Strains are colored by host and are indicated by the Host_GenBank Accession number_Strain_Country of isolation_Year of isolation.
