# Supplementary figures and images for: The Broad Host Range and Genetic Diversity of Mammalian and Avian Astroviruses
Source: Viruses. 2017 May 10;9(5):102. doi: 10.3390/v9050102 (PMC5454415; doi:10.3390/v9050102)

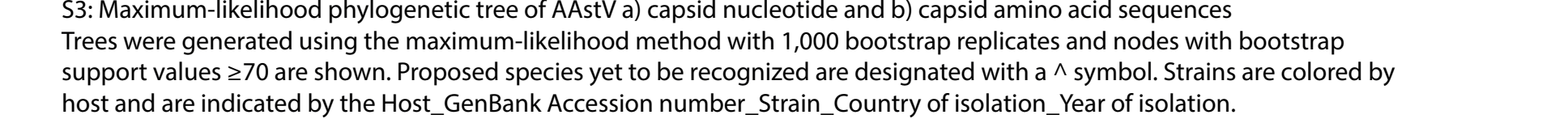

Supplement: Supplementary file 1 [file viruses-09-00102-s001.zip › viruses-190761_final_supplementary/FigureS3.pdf]
